# Varietas Delectat: Exploring Natural Variations in Nitrogen-Fixing Symbiosis Research

**DOI:** 10.3389/fpls.2022.856187

**Published:** 2022-04-11

**Authors:** Ting Wang, Benedikta Balla, Szilárd Kovács, Attila Kereszt

**Affiliations:** ^1^Eötvös Loránd Research Network, Biological Research Centre, Institute of Plant Biology, Szeged, Hungary; ^2^Doctoral School in Biology, University of Szeged, Szeged, Hungary

**Keywords:** symbiotic nitrogen fixation, rhizobium strains, legume ecotypes/cultivars, symbiotic incompatibility, partner dependent mutation manifestation

## Abstract

The nitrogen-fixing symbiosis between leguminous plants and soil bacteria collectively called rhizobia plays an important role in the global nitrogen cycle and is an essential component of sustainable agriculture. Genetic determinants directing the development and functioning of the interaction have been identified with the help of a very limited number of model plants and bacterial strains. Most of the information obtained from the study of model systems could be validated on crop plants and their partners. The investigation of soybean cultivars and different rhizobia, however, has revealed the existence of ineffective interactions between otherwise effective partners that resemble gene-for-gene interactions described for pathogenic systems. Since then, incompatible interactions between natural isolates of model plants, called ecotypes, and different bacterial partner strains have been reported. Moreover, diverse phenotypes of both bacterial mutants on different host plants and plant mutants with different bacterial strains have been described. Identification of the genetic factors behind the phenotypic differences did already and will reveal novel functions of known genes/proteins, the role of certain proteins in some interactions, and the fine regulation of the steps during nodule development.

## Introduction

Nitrogen is an essential macronutrient for plants and is required for the synthesis of nucleic acids, amino acids, and many other important metabolites. It is one of the most limiting elements for plant growth despite dinitrogen gas (N_2_) accounting for a large proportion (around 78%) of Earth’s atmosphere. Its strong chemical stability, however, makes it inaccessible for most organisms including plants, only certain prokaryotic microorganisms can fix nitrogen, i.e., to break the triple covalent bonds between the nitrogen atoms and produce ammonium (Vance, 2001).

Leguminous plants have the unique ability to grow in nitrogen-poor soils because they establish symbiosis (Suzaki et al., 2015) with a wide range of nitrogen-fixing Gram-negative α- and β-Proteobacteria collectively referred to as rhizobia (Masson-Boivin et al., 2009; Lindström and Mousavi, 2020). This interaction provides advantages for the participating partners. Legumes have access to reduced nitrogen, which they can metabolize, at the cost of energy and organic materials originating from photosynthesis. At the same time, bacteria are provided by a nutrient-rich environment in the symbiotic nodules formed on the roots or occasionally on the stems of the host plant (see later), where a much larger population of descendants than in soil can be established. As crop and pasture legumes can fix as much as 200–300 kg of nitrogen per hectare per year (Peoples et al., 1995), they have been an important element of crop rotation systems for a very long time and provide multiple benefits for agriculture sustainability (Stagnari et al., 2017). The ability of legumes to convert N_2_ into ammonia, as well as the first isolation and the morphological changes of rhizobia from and in nodules, were demonstrated in the 1880s, which was quickly followed by the market introduction of the first commercial *Rhizobium* inoculant (Nitragen) in 1895, almost 20 years earlier than performing industrial-scale ammonia synthesis by Carl Bosch (for a historical review, see Soumare et al., 2020). Since then, many inocula containing rhizobia for different legumes have been developed and commercialized to improve the yield of leguminous crops through symbiotic nitrogen fixation.

The development of the symbiosis between leguminous plants and rhizobia is a complex program including several interconnected developmental processes (bacterial infection and nodule organogenesis), multiple exchanges of signals (Gibson et al., 2008), and the activity and coordinated regulation of the expression of numerous genes (Mergaert et al., 2020; Roy et al., 2020). Legumes perceive the lack of nitrogen in soils and secrete flavonoids into the rhizosphere that are recognized as signals by rhizobia (Peters et al., 1986; Redmond et al., 1986) and—through their putative interaction with the NodD transcription factor (TF) proteins (Györgypál and Kondorosi, 1991; Györgypál et al., 1991; Peck et al., 2006), inducing the expression of nodulation (*nod*) genes. The proteins encoded by *nod* genes are essential for the synthesis and export of lipo-chitooligosaccharides called nodulation or Nod Factors (NFs) that have a core structure with 4-5 N-acetyl-glucosamine residues and an acyl chain conserved in all different species of rhizobia. Length and saturation level of the acyl chain, as well as the decoration of the backbone with several chemical modifications, such as methyl, acetyl, carbamoyl, or fucosyl groups are determined and mediated by enzymes encoded by the strain-specific *nod* genes and contribute to the specificity of the symbiosis between the partners (Dénarié et al., 1996; Long, 1996). Even in the absence of rhizobia (Truchet et al., 1991), the NFs induce quick ion fluxes through the membrane of root hairs (Felle et al., 1988), oscillations in calcium concentrations (calcium spiking) in the nuclei of epidermal cells (Ehrhardt et al., 1996), swelling and deformation of the root hairs as well as division of cortical cells (Catoira et al., 2000). The NFs are recognized by a membrane-anchored receptor complex and the perceived signal is transmitted through the so-called common symbiosis signaling (CSS) pathway, shared by another beneficial symbiosis that is established with arbuscular mycorrhizal fungi (Suzaki et al., 2015; Kronauer and Radutoiu, 2021) and, then, translated into gene expression changes by a network of transcription factors (Diedhiou and Diouf, 2018).

**FIGURE 1:**
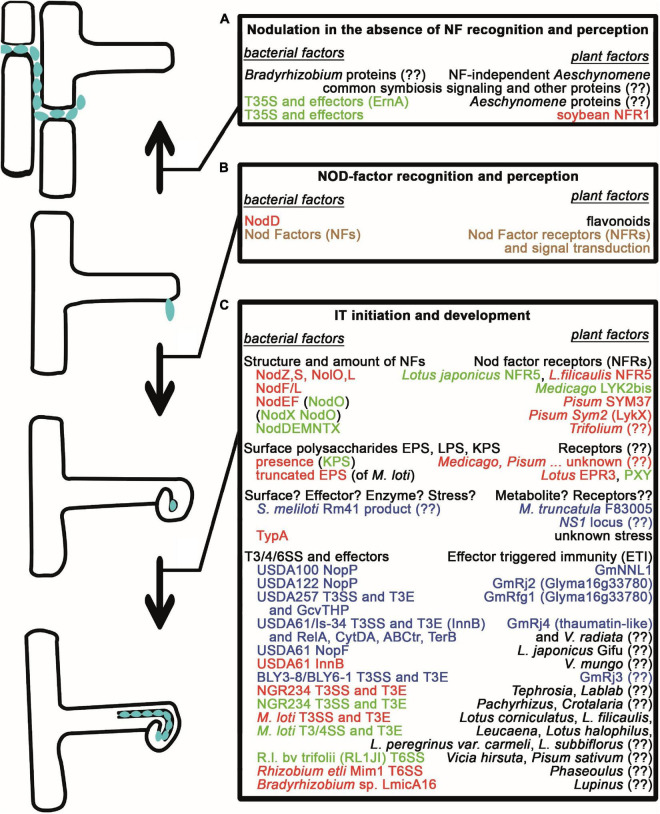
Natural variations affecting early nodule development and infection. **(A)** Nodule formation and intercellular infection in the absence of Nod factors and NF recognition/perception requires either an unknown mechanism or the activity of Type III secretion systems for the translocation of effector proteins in bradyrhizobia as well as the common symbiosis signaling pathway and other unknown proteins in *Aeschynomene* species and in soybean plants. **(B)** Nod Factor production of rhizobia and NF recognition/perception are required for the early symbiotic events such as root hair curling. **(C)** Initiation and progression of bacterial invasion through ITs are strictly controlled. The structure and amount of NFs determine whether NF receptors allow the initiation of IT development. Similarly, the presence of surface polysaccharides with correct structure is checked by the plants with help of receptors and unknown mechanisms. Unknown mechanisms and stress from the plants also might inhibit infection unless bacteria can deal with them, for example, by expressing the TypA stress protein. Bacterial effector molecules (Nops) resembling pathogen virulence factors transported into the plant cells and Effector Triggered Immunity often led to the restriction of infection, however, there are cases, when they have a positive effect on nodulation. Those bacterial macromolecules, whose lack or incorrect structure led to the arrest of the interaction with certain partners, and those plant proteins, which restrict the bacterial mutants or whose lack can be overturned, are shown in red. Plant molecules, as well as bacterial proteins/molecules, whose lack or presence (in parenthesis), that are able to overturn these defects, are shown in green. Those bacterial and corresponding plant proteins, that are responsible for incompatibility and a defect in either of them leads to compatibility, are shown in blue. If the compatibility needs both the plant and bacterial factors, the proteins are shown in brown. (??) denotes that the given factor has not been identified yet.

In the presence of NF-producing rhizobia and after the original electrophysiological changes, bacteria enter the root and then, invade the cells of the developing nodules (Figure 1). Depending on the interaction, there are two main ways for rhizobia to enter root tissues (Ibáñez et al., 2017): (i) For intercellular invasion, bacteria cross the epidermal layer between neighboring root hair cells or root hair and epidermal cells or through cracks/fissures, and spread through the cortex between cell walls or intercellular air spaces or by a progressive collapse of the invaded cells; (ii) In the model legume plants (*Medicago truncatula* and *Lotus japonicus*), as well as in most crop plants (such as soybean, bean, and pea), rhizobia invade roots through the transcellular infection threads (ITs), tubular structures that guide bacteria into the inner tissues of the nodule. In this latter mode, NF production by attached rhizobia induces continuous re-orientation of the root hair growth resulting in a shepherd’s crook-like curled root hair, which forms a so-called infection pocket and surrounds bacteria that establish a microcolony (Gage, 2004). In the infection pocket, the IT is initiated by cell wall degradation and invagination of the root hair membrane (Murray, 2011), and then, it extends by polar growth toward the base of the root hair cell, enters the cortical cell layers until it reaches the new cells produced by the nodule primordium. The IT polar growth requires the coordinated and dynamic action of several proteins that determine membrane domains, polarity, or involvement in the rearrangement of the cytoskeleton or the regulation of NF levels (Tsyganova et al., 2021). On the rhizobial side, the initiation and growth of ITs required from the rhizobia, which are topologically in the extracellular space and multiply in the growing ITs, the adaptation to the specific osmotic, pH, and ionic environment of ITs (Dylan et al., 1990; Putnoky et al., 1998), the regulation of NF levels (Malolepszy et al., 2018) and correct production of surface polysaccharides (López-Baena et al., 2016), such as extracellular polysaccharide (EPS), K-antigen capsular polysaccharide (KPS), or lipopolysaccharide (LPS). In *L. japonicus*, a receptor structurally similar to the NF receptors monitors, whether the EPS of the symbiotic bacteria has the correct structure, i.e., it prevents bacterial entry if *Mesorhizobium loti* produces truncated EPS, but it allows infection of bacteria that produce wild-type or no EPS (Kawaharada et al., 2015). In contrast, the *Sinorhizobium meliloti* strains that are defective in the production of the succinoglycan EPS are not able to infect *Medicago* roots (Leigh et al., 1985), unless they produce a second exopolysaccharide (galactoglycan EPS II) or KPS (Glazebrook and Walker, 1989; Putnoky et al., 1990; Pellock et al., 2000). In other legumes, such as *Glycine* or *Phaseolus* species, the correct structure of LPS is required for the successful infection program (Carlson et al., 1987; Stacey et al., 1991; Margaret et al., 2013).

**FIGURE 2:**
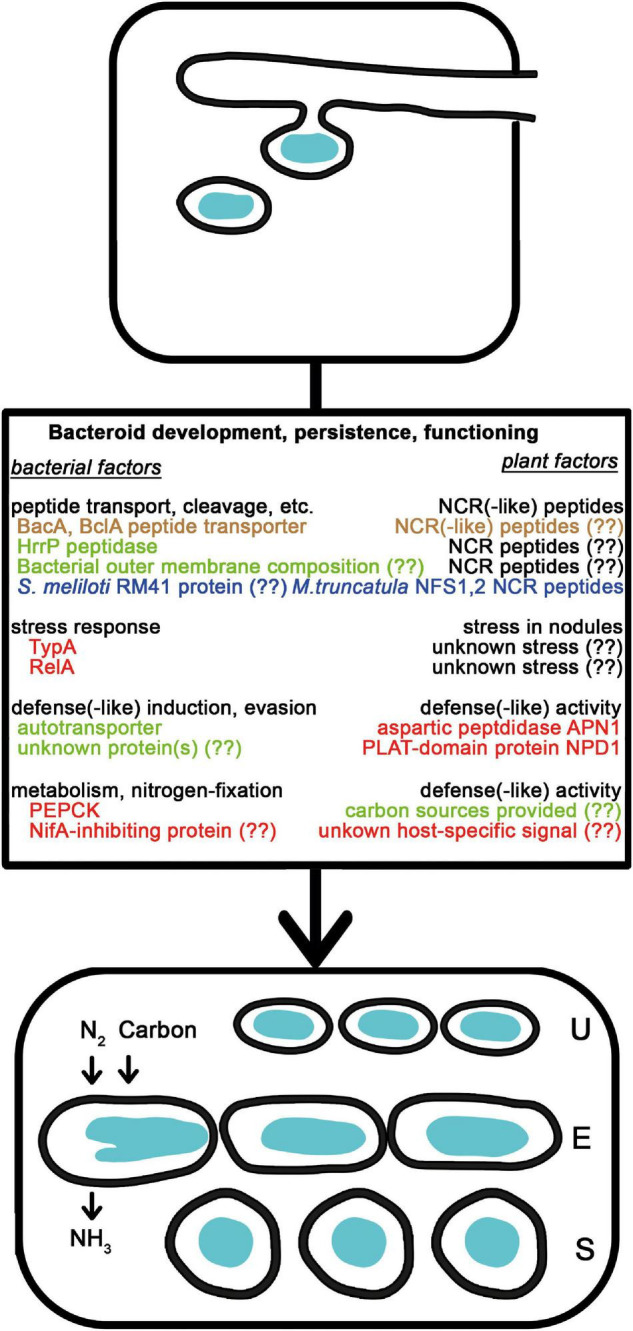
Natural variations affecting bacteroid development, persistence, and functioning. After the release of rhizobia from the ITs, bacteria differentiate into nitrogen-fixing bacteroids of unmodified (U), elongated (E), or spherical (S) morphotypes. The NCR peptides affecting bacterial membrane and intracellular functions are delivered into bacteria by peptide transporters and contribute to the terminal differentiation of E and U morphotype bacteroids. Bacterial outer membrane composition, peptidases and independently evolved transporters affect the success of bacteroid development. The NCR peptides are also involved in strain discrimination. The level of stress and the available carbon sources in the plants affect how bacteria with mutations in stress related genes or carbon metabolism perform. Bacteria also might induce and endure plant defense reactions and sense signals from certain plants that affect gene expression. Colors are the same as in Figure 1.

In the newly formed differentiating nodule cells, individual bacteria are released from the ITs through an endocytosis-like process (Figure 2) and become surrounded by a membrane of host origin called peribacteroid or symbiosome membrane (Roth and Stacey, 1989). These organelle-like structures, called symbiosomes, divide to fill the cytoplasm of the nodule cells and the rhizobia, thus, differentiating into their nitrogen-fixing form called bacteroids (Vasse et al., 1990). The parallel differentiation of bacteria and plant cells is accompanied by drastic physiological, metabolic, and gene expression changes. The new cells formed by the nodule meristem and being infected by rhizobia complete multiple cycles of endoreduplication resulting in enlargement of nuclear and cell volumes (Foucher and Kondorosi, 2000), change their expression profile in multiple waves (Maunoury et al., 2010) and adjust their metabolism and the cellular environment for nitrogen fixation (Udvardi and Poole, 2013). To protect the oxygen-sensitive nitrogenase enzyme and at the same time, to support bacterial respiration energizing nitrogen fixation, the free oxygen concentration is kept at a very low but constant level within the infected nodule cells. The low free oxygen concentration is achieved by a physical barrier in the outer cortical layer of the nodule and massive production of the oxygen-binding leghemoglobin protein (Rutten and Poole, 2019). This low oxygen concentration regulates the production of bacterial proteins that are involved in the reduction of nitrogen, in the metabolism and transport of the fixed nitrogen, as well as in the exchange of metabolites between the bacterial and plant cells (Terpolilli et al., 2012; Udvardi and Poole, 2013).

After reaching its peak, the nitrogen fixation in the nodule cells declines, and this is accompanied by the decrease of leghemoglobin concentration and nitrogenase activity by morphological changes of the cells, or lifestyle of the rhizobial population present. This complex and programmed process called nodule senescence is a normal stage of the symbiosis, however, it can also be induced by the transition from vegetative to the reproductive stage of plant development or by adverse environmental and physiological conditions (Zhou et al., 2021).

Based on their development and morphology that is determined by the plant, both the nodules and the bacteroids can be divided into two major types (Ferguson et al., 2010; Alunni and Gourion, 2016). Indeterminate nodules on the roots of, for example, the *Medicago*, *Pisum*, *Coronilla*, *Leucaena*, and *Amorpha* species have a cylindrical shape resulting from the activity of a persistent meristem, which continuously produces new cells. These new cells become infected with bacteria and develop into nitrogen fixation tissue. As a consequence, indeterminate nodules keep increasing in size and have a gradient of developmental stages recognized as meristematic (Zone I), infection (Zone II), differentiation (Interzone II-III), nitrogen fixation (Zone III), and in older nodules, the senescence (Zone IV) zones. In contrast, determinate nodules that form on legume species from, for example, the *Lotus*, *Glycine*, *Phaseolus*, *Aeschynomene*, or *Arachys* genera are spherical and the cell population in the inner tissues is relatively homogenous and does not form a developmental gradient. During the formation of determinate nodules, the meristem is not persistent, it ceases to divide at an early stage, and further nodule development takes place more-or-less synchronously.

Independently from the nodule morphology, the differentiation and the fate of bacteria also depend on the plant host (Mergaert et al., 2006): Bacteroids in the nodules of *Lotus*, *Glycine*, *Phaseolus* or *Leucaena* are similar to the free-living cells in shape, size, DNA content and retain their cell division capacity. In contrast, bacteroids in the nodule cells of *Medicago*, *Pisum*, *Aeschynomene* or *Arachys* species have increased size, membrane permeability and DNA content, different—spherical or elongated-(branched)—shape compared to their free-living siblings. These nodule bacteria also lost their cell division capacity, thus, they are considered terminally differentiated bacteroids (Alunni and Gourion, 2016). The ability to impose terminal bacteroid differentiation on the bacterial partner appeared at least five times during the evolution of the *Papilionoideae* legumes indicating a possible fitness benefit for the plant (Oono et al., 2010). Using bacteria, which can establish symbiosis with plant’s hosts governing different bacteroid fates, it was shown that terminally differentiated bacteroids have increased symbiotic efficiency as compared to unmodified bacteroids (Oono and Denison, 2010). Moreover, even the level of terminal differentiation correlates with nitrogen fixation efficiency, as in nodules of different *Aeschynomene* species, highly polyploid spherical bacteroids are more efficient than the elongated ones with lower ploidy level (Lamouche et al., 2018, 2019). Terminal bacteroid differentiation is induced by plant-derived molecules, termed as nodule-specific cysteine-rich (NCR and NCR-like) peptides (Mergaert et al., 2003; Van de Velde et al., 2010; Czernic et al., 2015), which are produced almost exclusively in the infected cells of nodules (Guefrachi et al., 2014) solely by those plants, which impose terminal bacteroid differentiation on their symbiont. The number and type of NCR peptides in the Inverted Repeat Lacking Clade (IRLC) of *Papilionoideae* legumes are highly variable and correlate with the morphotype of the bacteroids in the nodules of these species (Montiel et al., 2016, 2017). The NCR peptides were shown to interact with several bacterial proteins in *M. truncatula*, thus, affecting, for example, the transcription, translation, and cell cycle regulation (Tiricz et al., 2013; Farkas et al., 2014; Penterman et al., 2014). Despite their high number (over 700 genes in *Medicago*) and putatively redundant functions, individual NCR peptides were proven to be required for bacteroid development and persistence (Horváth et al., 2015; Kim et al., 2015).

## Natural Variations Superimposed on General Nodule Developmental Pathways

In general, if the symbionts possess the above-reviewed tool kits, they can establish symbiosis with the partners in their cross-inoculation group to reduce atmospheric nitrogen and to support plant growth. However, it was recognized by early investigations performed mainly on crop plants that there exist symbiotic incompatibilities (i.e., no nodule formation or no nitrogen fixation) among symbionts that form effective interactions with other cultivars, or strains of the partner species (Bergersen and Nutman, 1957; Caldwell, 1966; Melino et al., 2012). It was also shown that when compatible interactions lead to nitrogen fixation, their efficiency, i.e., the plant benefit derived from the symbiosis is highly variable (Snyman and Srijdom, 1980; Terpolilli et al., 2008; Kazmierczak et al., 2017). To further complicate the picture, mutations may have different effects on the interaction depending on the partners investigated [for example (Wilson et al., 1987; Osteras et al., 1991; Rodpothong et al., 2009)].

### Natural Variation in the Stringency Requirements of Nodulation Factor Induced Processes

#### Nodulation Factor Recognition in *Medicago*

It was recognized early after the discovery of NFs that the induction of root hair deformation and cortical cell division does not require the full and proper decoration of these signal molecules in contrast to root hair curling, as well as the initiation and growth of ITs (Ardourel et al., 1994). IT development in alfalfa root hairs was strongly inhibited by the *nodL* and *nodEF* mutants of *Sinorhizobium meliloti* lacking the O-acetylation and N-acylation with specific fatty acids, respectively, at the non-reducing end of NFs that was more pronounced with the double mutant. These observations led to the hypothesis that different receptor forms, i.e., less stringent signaling receptors for the initiation of root hair deformation and cortical cell division, and the entry receptors with more rigorous requirements for bacterial infection regulate the nodulation process.

Similar to *M. sativa*, most *M. truncatula* accessions form no nodule with the *nodF/nod*L double mutant *S. meliloti* strains, but *M. truncatula* ssp. *tricycla* R108 establishes an effective nitrogen-fixing symbiosis with the mutant as with the wild-type strain (Luu et al., Under review^1^). As the NF receptor proteins NFP and LYK3 (Arrighi et al., 2006; Smit et al., 2007) in ecotype Jemalong, and the NFP protein in R108 (Feng et al., 2019) were shown to be essential for NF perception and nodulation, the genomic sequence of R108 around the *LYK3* gene was analyzed in more detail. Between *LYK2* and *LYK3*, an additional gene designated as *LYK2bis*, which is a chimera of the two neighboring genes, was identified in R108 that could not be found in any other *M. truncatula* accessions with a sequenced genome. Using loss-of-function (*lyk2bis* mutant in R108) and gain-of-function (transforming *LYK2bis* into Jemalong) approaches, it was proven that the LYK2bis protein enables R108 to be nodulated and infected by the *nodF/nodL* mutant.

#### Variations in the *Rhizobium leguminosarum* Nodulation Factor Structure Requirements

The *nodE* gene of *Rhizobium leguminosarum* was also shown to be a determinant of host specificity of the biovars and mutations in this gene’s generally decreased nodulation on pea (Surin and Downie, 1989). Interestingly, the *nodE* mutation in *R. leguminosarum* bv. *viciae* almost blocked the nodulation on certain pea lines but affected less severely the other accessions. Genetic analysis revealed that *nodE*-dependent nodulation is associated with a haplotype (Li et al., 2011) of the *PsSym37* gene coding for a LysM-type receptor kinase, which is closely related to the LjNFR1/MtLYK3 Nod Factor receptors, and is essential for infection-thread initiation in pea (Zhukov et al., 2008). Wild isolates—commonly referred to as Afghan peas—from the Middle East (Afghanistan, Iran, Turkey, Israel, Uzbekistan, and Tajikistan) and known as the center of origin for peas, cannot be nodulated by rhizobia collected in Europe, but only by strains isolated from soils from the same region (Lie, 1984). In Afghan peas, cortical cell divisions are initiated upon induction by European *R. leguminosarum* bv. *viciae* strains but IT formation and bacterial invasion are blocked. This inhibition can be overcame either by growing the plants at elevated temperature (Kozik et al., 1995) or by the production of the NodX protein in rhizobia that adds an O-acetyl group to the C6 carbon of N-acetylglucosamine residue at the reducing end of the pentameric NF (Götz et al., 1985; Firmin et al., 1993). Genetic analysis of the locus called *sym2*, which is responsible for the incompatibility of Afghan peas, added a level of complexity. The *sym2^A^* allele of Afghan peas is dominant over the *sym2^C^* alleles of cultivated peas when *R. leguminosarum* bv. *viciae* strain PRE is used as inoculum, however, the dominance is changed when strains 248 or PF2, producing higher amount of Nfs, are applied (Kozik et al., 1995). Despite the long history of research on the specificity of Afghan peas, no direct evidence, such as changing the specificity of a cultivated line by transformation, about responsible for the strict partner selection has emerged. However, several genetic and bioinformatics data indicates that another LysM-type receptor kinase, termed PsLykX (for *P. sativum* LysM kinase eXclusive), probably interacting with the pea NFR1 (PsSym10) receptor may determine the trait (Sulima et al., 2017, 2019; Solovev et al., 2021). Interestingly, the *nodO* gene coding for a secreted protein, which was shown to form ion channels in membranes (Sutton et al., 1994), can compensate both for the *nodE* mutation (Walker and Downie, 2000) and partially or fully for the absence of the *nodX* gene in strain 248 on plants carrying the *sym2^A^* allele in homozygous or heterozygous forms, respectively (Geurts et al., 1997). The non-nodulating phenotype of the *nodO* mutant of strain 248 on the heterozygous plants, as compared to nodule formation by wild-type bacteria, might explain the strain-specific differences regarding the dominance of the *sym2* alleles. It is possible that the genome of strain PRE does not code for a functional NodO protein for compensation. It is hypothesized that NodO might function either to bypass the NF receptor activity or to amplify a weak signal originating from the interaction of the not fully compatible NFs and receptors.

Similar variability in NF recognition and sensitivity might exist in *Trifolium* species. *R. leguminosarum* bv. *trifolii* strain TA is not able to nodulate *T. subterraneum* cv. Woogenellup at 22°C because of the arrest of IT development but forms an effective symbiosis with this cultivar at 28°C (Lewis-Henderson and Djordjevic, 1991a). This trait is determined by the recessive alleles of a gene named *rwtl* (resistance of Woogenellup to strain TA1). The nodulation deficiency of strain TA1 is overcome by mutations in genes that affect the structure (*nodE* and *nodX*), the amount (*nodD*, *nodM*, *nodN*, and probably, the negatively acting *csn-1* (for *cultivar-specific nodulation*) of unknown identity and function), or transport (*nodT*) of Nod Factors (Lewis-Henderson and Djordjevic, 1991b).

#### Nodulation Factor Recognition in *Lotus* Species

The most numerous and most detailed investigations of NF recognition and perception have been performed in *Lotus* species, mostly in *L. japonicus*, serving as a model for determinate nodule development, in symbiosis with *Mesorhizobium loti*. This rhizobium produces mainly pentameric NFs with an acetylated fucosyl residue at the C6 carbon of the reducing sugar, a carbamoyl group, and a N-methylated 18:1 acyl chain at the C4 and C2 carbons, respectively, of the non-reducing sugar (López-Lara et al., 1995). The symbiotic capability of *M. loti* R7A mutants that fail to synthetize acetyl-fucosylated NFs depends on the *Lotus* species serving as partner (Rodpothong et al., 2009). Nodule formation by a *nolL* mutants failing to add the acetyl modification to the fucosyl moiety is delayed on all four *Lotus* species (*L. japonicus*, *L. corniculatus*, *L. filicaulis*, and *L. burttii*) tested. Moreover, IT formation in *L. filicaulis* is also arrested. Defects in the synthesis (in the *nolK* and *noeL* mutants) and transfer to the NF backbone (in the *nodZ* mutant) of the fucosyl residue prevent infection not only in *L. filicaulis* but also in *L. corniculatus*, revealing different stringency requirements in the *Lotus-Mesorhizobium* cross-inoculation group. An engineered symbiont of *Lotus* called strain DZL and was created by expressing an inducer-independent NodD protein, as well as the NodZ fucosyl-transferase and the NolL acetyltransferase in *R. leguminosarum* bv. *viciae* (Pacios Bras et al., 2000), produces NFs that only differ from those of *M. loti* in the decorations of the non-reducing sugar, i.e., the acyl chain on the C2 carbon is not methylated, the C6 carbon is not carbamoylated but the C5 carbon is acetylated. This strain effectively nodulated *L. japonicus* but failed to infect *L. filicaulis*. It was shown by domain and amino acid swaps between the Nfr receptors of the two species that a single amino acid difference in the M2 domain of Nfr5 is responsible for the differential recognition of NFs (Radutoiu et al., 2007).

#### Summary

Legumes rigorously choose their rhizobial partners by recognizing the chemical structure of the first signal molecules, the Nod Factors, via the activity of the NF receptors. Structural variations and extension of the NF receptor repertoire might both strengthen (NodX requirement by SYM2) or weaken (LYS2bis) the stringency of the identification process, which is also affected by the amount of NFs and temperature.

### Natural Variation in Surface Polysaccharide Requirements for Infection

In the indeterminate nodule forming rhizobia, an efficient infection (thread development) requires the production of exopolysaccharides (EPSs), the lack of EPS resulting in no IT formation or in ITs aborted in the root hairs (Skorupska et al., 2006). In *S. meliloti* strain Rm41, however, EPS-deficient (*exoB* mutant) bacteria induce the formation of infected, nitrogen-fixing nodules on alfalfa and this phenotype is associated with its ability to produce a strain-specific K-antigen (KPS), a polymer of a disaccharide repeating units composed of glucuronic acid and N5-β-hydroxybutyryl-N7-acetyl-5,7-diamino-3,5,7,9-tetradeoxynonulosonic acid (pseudaminic acid) on the surface (Putnoky et al., 1990; Kereszt et al., 1998; Reuhs et al., 1998). Interestingly, if the *lpsZ* (*rkpZ*) gene, found in the Rm41 strain-specific *rkp-3* gene cluster (Kiss et al., 2001), responsible for the synthesis of the pseudaminic acid precursor, as well as for the export of the KPS, is introduced into an EPS-deficient mutant of strain 1,021 producing a structurally different KPS, the transconjugant forms effective symbiosis with alfalfa (Williams et al., 1990). The LpsZ protein determines (decreases) the polymerization level of the KPS in both strains and enables the formation of the symbiotically efficient molecular weight form (Reuhs et al., 1995). The KPS of strain Rm41 can complement for the absence of EPS, not only on alfalfa (*M. sativa*), but also on other *Medicago* (*M. media* and *M. varia*) and *Melilotus* (*M*. *albus* and *M. officinalis*) species tested (Putnoky et al., 1990), however, not on *M. truncatula* (Hozbor et al., 2004; Liu et al., 2014). In *S. meliloti* strain 1,021, a third polysaccharide, the galactoglucan EPS II with a disaccharide repeating unit of a β-(1-3)-linked acetylated glucose and succinylated galactose can contribute to the infection process by replacing the succinoglycan EPS I during nodulation of *M. sativa* (Glazebrook and Walker, 1989). However, EPS II cannot function in place of EPS I on other investigated hosts such as *M. coerulea*, *M. truncatula*, *Melilotus albus*, and *Trigonella foenum-graecum*.

Determinate nodule formation and infection require the correct structure of LPS on the bacterial surface, while the production of exopolysaccharides by rhizobia infecting these nodules seems to be not important. In *Lotus corniculatus* and *L. japonicus* Gifu, however, bacteria, which do not produce EPS, can establish as effective symbiosis as the wild-type strain, whereas mutants affected in mid or late biosynthetic steps (e.g., *exoU*) and produce truncated form of the polysaccharide induced uninfected nodule primordia (Kelly et al., 2013). In contrast, *L. japonicus* MG20 is less stringent in its selection because it forms nitrogen-fixing nodules with mesorhizobia that are both producing no or truncated EPS. The incompatibility in *L. japonicus* Gifu is mediated by the LysM-type receptor EPR3 (Kawaharada et al., 2015), recognizing the diffusible octasaccharide monomer of EPS, not only from *M. loti*, but also from *R. leguminosarum* and *S. meliloti* (Wong et al., 2020). The PXY leucine-rich repeat receptor-like kinase in *L. japonicus* MG20, which also regulates stem vascular development, was identified by quantitative trait locus sequencing (QTL-seq) as a casual component of the differential and less stringent *exoU* response (Kawaharada et al., 2021).

The *S. fredii* strain HH103 establishes symbiosis with a wide variety of legumes forming indeterminate and determinate nodules (Margaret et al., 2011). Infection of the determinate nodules of soybean and pigeon pea (*Cajanus cajan*) by this bacterium necessitates the production of the 5-acetamido-3,5,7,9-tetradeoxy-7-(3-hydroxybutyramido)-L-glycero-L-manno-nonulosonic acid homopolymer K-antigen, however, KPS-deficient mutants of HH103 induce infected and nitrogen-fixing determinate nodules on cowpea (Parada et al., 2006; Hidalgo et al., 2010).

#### Summary

The presence of certain polysaccharides on the bacterial surface is essential for the infection process monitored by a currently unknown mechanism in the plants. Certain bacteria are able to produce alternative polysaccharides to compensate for the absence of the generally used ones, however, this compensation might not be effective on all hosts. Moreover, not only the presence, but the correct structure, for example, of the EPS is checked by another mechanism.

### Other Natural Variations Affecting Bacterial Infection

Large collections of legume hosts and bacteria isolated from nodules developed on plants in natural habitats have allowed the establishment of cross-inoculation groups and investigation of natural variations within cross-inoculation groups and species. Investigations, for example, with the model legumes *L. japonicus* and *M. truncatula* and/or species closely related to them, revealed large variations in symbiotic compatibility and efficiency (Snyman and Srijdom, 1980; Crook et al., 2012; Gossmann et al., 2012; Granada et al., 2014; Liu et al., 2014; Lorite et al., 2018). For example, experiments using a number of *Medicago* species and *M. truncatula* accessions in combination with different *S. meliloti* isolates revealed tremendous variation in nodulation capacity and nitrogen fixation specificity between different genotype-rhizobial combinations, 40–50% of all host-strain pairs resulted in an ineffective symbiosis (Crook et al., 2012; Liu et al., 2014). Detailed characterization accompanied with the identification of the genes and alleles determining these symbiotic variations beyond soybeans’ incompatibility (see later) has been very rare.

Liu et al. (2014) reported that *S. meliloti* strain Rm41 induced root hair curling and nodule primordium formation but failed to infect the roots of *M. truncatula* ecotype F83005.5, although both partners have the genetic capacity for nitrogen fixation. The infection process is arrested at the microcolony stage, either no or occasionally, aberrant ITs not entering the cortex can be observed on the roots. This phenotype is similar to the ones induced by EPS-deficient mutants of *S. meliloti* or by *M. loti* producing truncated EPS or by rhizobia incompatible with certain genotypes of soybean. This dominant trait in *M. truncatula*, however, must be independent of these bacterial effectors. EPS recognition can be ruled out because the strain produces wild-type EPS and its EPS-deficient derivatives cannot establish effective symbiosis with other ecotypes of the plant either. The possibility of effector-triggered immunity (ETI) responsible for the soybean incompatibilities (see later) can also be excluded because the genome of strain Rm41 does not code for any Type III, Type IV, and Type VI secretion systems implicated in ETI.

An *R. leguminosarum* strain (termed Norway) isolated from *L. corniculatus* nodules shows host-genotype specific differences when inoculated on wild-type plants of *Lotus* species and pea cultivars. It induced no nodules on *L. japonicus* Gifu and *L. filicaulis*, *L. japonicu*s MG20 formed bumps and occasionally, very small and infected nodules, while *L. japonicus* Nepal showed broadened elongated infection zones. On *L. glaber*, the strains provoked the development of swellings and tumor-like structures, while *L. burtii* developed normal-sized and infected but inefficient nodules. The strain-induced ITs on *Pisum sativum* cv. Sparkle without the formation of nodule(-like) structures, whereas pea cultivar Little Marvel and *Latyrus sativus* plants had ineffective nodules (Gossmann et al., 2012; Liang et al., 2019). In *Lotus* species, the strain induced early and strong induction of a symbiosis-specific gene but no ITs, and rather intercellular accumulation of the bacteria through epidermal cracks could be observed. The strain could invade intact nodule cells where it formed symbiosomes, however, these infected cells exhibited the signs of early senescence (Liang et al., 2019). As the strain seems to possess all the genetic repertoire required for and still fails in the establishment of an effective sysmbiosis (Liang et al., 2018), it will be interesting to find one or more compatible hosts and determine which factors in the bacteria and plants cause the incompatibilities.

#### Summary

The incompatible interactions arrested at the infection stage indicate the presence of additional checkpoints beyond NF and polysaccharide recognition.

### Bacterial Effector Molecules and Plant Immunity Affecting Compatibility

Bacteria secrete proteins and other (macro)molecules to modulate their interactions with the environments, especially when they are interacting with eukaryotic host organisms. In the case of Gram-negative bacteria, secretion requires translocation across both the inner and the outer membranes, and several different molecular machines have been elaborated for this purpose. Many proteins secreted by pathogens and symbionts are aimed to enter the host cells to modify the physiology of the partner, and, thus, several secretion systems include an apparatus to translocate proteins across the plasma membrane of the host also (Tseng et al., 2009). In this context, it is not surprising that rhizobia are also equipped with certain or all types of effector delivery machinery that may have special roles in their interactions with different legume partners.

#### Microbe-Associated Molecular Patterns and Effector-Triggered Immunity, Type III Secretion System Effectors, and Symbiotic Compatibility

Plants developed a multilayered defense system against microbes that acts both locally and systematically (Ngou et al., 2021) and its elements are also important during the interaction of legumes and rhizobia (Cao et al., 2017). The first line of defense detects the presence of microorganisms *via* the activity of receptors recognizing microbe/pathogen-associated molecular patterns (MAMPs/PAMPs), which are conserved motifs present on essential components of a microbe/pathogen. The binding of MAMPs by these pattern recognition receptors (PRRs) induces rapid changes in the cell—as can be observed upon the recognition of NFs—and leads to MAMP/PAMP-triggered immunity (MTI/PTI). Interestingly, rhizobial MAMPs investigated, so far, such as flagellin or LPS, differ from, for example, those of plant pathogenic or enteric bacteria and do not induce MTI (Tellström et al., 2007; Lopez-Gomez et al., 2012). The legume hosts also evolved recognition mechanisms to distinguish beneficial and harmful microorganisms. *L. japonicus* and *M. truncatula* have LysM pattern-recognition receptors that are related to the NF receptors to separate the perception of chitin oligomeric microbe-associated molecular patterns from the perception of NFs by the NFR1/NFR5 receptor complex (Bozsóki et al., 2017). Another LysM receptor in *L. japonicus*, EPR3 distinguishes *M. loti* cells producing no or normal EPS from those that produce a truncated one with a pentasaccharide repeat instead of an octasaccharide repeat (Kawaharada et al., 2015). Although MTI/PTI at the cell surface is very effective, microbes evolved virulence factors and apparatus for their delivery to the host cells to modify or attenuate the original immune responses. The type III secretion system (T3SS) of Gram-negative bacteria including pathogens and symbionts is a complex multiprotein secretion apparatus that actively exports effector proteins (T3 effectors) with diverse biochemical activities (Schreiber et al., 2021) through the lumen of these tubular structures and directly into the eukaryotic host cells. In rhizobia, proteins that are either extracellular components of or secreted by the T3SS apparatus are termed as Nops, nodulation outer proteins, and are produced upon NF induction (Staehelin and Krishnan, 2015). The T3 effector proteins can be detected by intracellular receptors of the plant immune system that are usually highly specific in the effectors they recognize, an observation that led to the gene-for-gene hypothesis (Flor, 1971). The recognition of the effector results in a very robust immune response called Effector Triggered Immunity (ETI), which is often culminated in programmed cell death called hypersensitive response to halt the spread of the pathogen.

##### Classical Resistance and Defense Proteins Determine Symbiotic Incompatibility in Soybeans

The incompatibilities between soybean cultivars and specific rhizobium strains that have been studied and described as gene-for-gene interactions for a long time (Hayashi et al., 2012) seem true to be determined by ETI. Four dominant genes of soybean, *Rj2* (Caldwell, 1966), *Rj3* (Vest, 1970), *Rj4* (Vest and Caldwell, 1972), and *Rfg1* (Trese, 1985) were described to prevent nodulation with certain soybean-nodulating strains belonging to the *Bradyrhizobium* and *Sinorhizobium* genera. The block of these interactions takes place just after the first initial steps. Root hair deformation and/or curling and cortical cell division can be observed although in a lower number than in compatible interactions, but ITs and nodule meristems are not formed (Sadowsky et al., 1995; Yasuda et al., 2016). Analyses of rhizobial mutants have revealed the important role of T3 effectors in the determination of incompatibility with all the *Rj2*, *Rj3*, *Rj4*, and *Rfg1* soybeans because mutants defective in the T3SS, or the production of certain effectors, could form functional nodules on the roots of their incompatible hosts (Meinhardt et al., 1993; Tsukui et al., 2013; Faruque et al., 2015; Tsurumaru et al., 2015; Shobudani et al., 2020; Ratu et al., 2021a).

The *Rj2*, *Rj4*, and *Rfg1* genes of soybean were identified by map-based cloning with surprising results. The *Rj4* gene codes for a thaumatin-like protein (TLP), which belongs to the PR-5 family of pathogenesis-related proteins (Tang et al., 2016), considered as effectors to biotic (for example, fungal attack) and abiotic (for example, osmotic shock) stresses. It was most probably evolved by recent local gene duplication and diversification because both *Rj4* and *rj4* plants harbor a gene coding for another thaumatin-like protein, which is highly similar to Rj4 (only 13 amino acid differences between the two proteins of 296 residues) but does not cause incompatibility with *B. elkanii* strain USDA61, while the second gene causing the incompatibility is present only in the *Rj4* plants. The fact that the Rj4 protein is not a classical receptor protein is surprising and it will be intriguing to understand how this thaumatin-like protein is involved in ETI to regulate bacterial infection.

Another surprising and interesting result was that the allelic variants of the same gene, *Glyma16g33780* coding for a toll-interleukin receptor/nucleotide-binding site/leucine-rich repeat (TIR-NBS-LRR) resistance (R) protein are responsible for the *Rj2* and *Rfg1* incompatibilities (Yang et al., 2010). It was shown that a single amino acid difference, isoleucine versus arginine at amino acid position 490 after the NBS domain in the *Rj2* and *rj2* alleles, respectively, determines symbiotic (in)compatibility (Sugawara et al., 2019). Polymorphism at five amino acids in and after the sixth LRR domain of this R protein differentiates the *Rfg1* and *rfg1* alleles (Yang et al., 2010), however, systematic investigation of these differences to reveal the role of the individual residues has not been conducted yet. All four possible alleles (*rj2*/*rfg1*, *Rj2*/*rfg1*, *rj2*/*Rfg1*, and *Rj2*/*Rfg1*) of the gene were constructed and shown to determine the expected compatibility profile when transformed into a non-restrictive (*rj2*/*rfg1*) soybean (Fan et al., 2017). A genome-wide association study (GWAS) to identify natural variants in key loci that regulate the compatibility between soybean and rhizobia, using Chinese landraces and improved cultivars and *B. diazoefficiens* strain USDA110 as inoculant, pinpointed and confirmed the *Glyma.02G076900* gene termed *G. max Nodule Number Locus 1* (*GmNNL1*) coding for another TIR–NBS–LRR receptor protein and carrying a SINE transposon in the compatible plants as the determinant of the incompatibility (Zhang et al., 2021).

##### Role of the Type III Secretion System and Nodulation Outer Proteins in Symbiotic Incompatibility With Soybeans

As for the bacterial side, the T3 effector NopP protein of strains USDA122 and USDA110 were shown to be responsible for the incompatibility with *Rj2* and *GmNNL1*-positive soybeans, respectively. Interestingly, if only three amino acids of USDA122 NopP are replaced by those of the compatible strain USDA110, the compatibility with *Rj2* cultivars is restored (Sugawara et al., 2018). Such specificity has not been identified between the *Rfg1* allele-encoded protein and any of the T3 effectors in strain USDA257. Lack of synthesis and/or secretion of the T3 effectors leads to compatibility with soybeans carrying the *Rfg1* allele (Meinhardt et al., 1993) indicating that classical ETI affects compatibility. Moreover, mutations disrupting the *gcvTHP* operon coding for the elements of the glycine cleavage system enables strain USDA257 to form nitrogen-fixing nodules on *Rfg1* soybeans (Lorio et al., 2010). The glycine cleavage system is involved in the generation of C1 units in the form of N^5^, N^10^-methylene tetrahydrofolate, that is used in a variety of biochemical reactions, including the synthesis of purines, histidine, thymine, and methionine as well as the formylation of tRNA^fMet^ already loaded with methionine. The *gcvTHP* mutations do not affect the synthesis and secretion of the Nop proteins in general, thus, further studies are required to elucidate whether a putative decrease in the C1 pool has a direct or indirect role. This putative decrease might affect, for example, the T3SS/T3 effectors or decrease the amount of the innate immune system activating formyl-methionine containing oligopeptides released by damaged bacteria as described in mammalian systems (Zhang et al., 2010).

The incompatibilities of *B. elkanii* strain USDA61 and *B. japonicum* strain Is-34 with *Rj4* soybeans are also dependent on the T3SS because these hosts form an effective symbiosis with the strains if their T3SS systems are non-functional (Faruque et al., 2015; Tsurumaru et al., 2015). Moreover, mutations in four genes coding for proteins (the RelA ppGpp synthetase; a cytosine deaminase; a TerB family tellurite resistance protein; a substrate-binding protein of an ABC transporter) that are not T3E effectors but might affect T3SS synthesis and/or function also restored compatibility (Nguyen et al., 2017). Indeed, it was recently shown that ppGpp synthetase deficient bradyrhizobia are not able to activate the T3SS (Pérez-Giménez et al., 2021).

##### Type III Secretion System and Nodulation Outer Proteins Can Both Restrict and Extend the Host-Range

The Nop proteins are indeed “double-edged swords” of rhizobia (Staehelin and Krishnan, 2015) because they can restrict nodulation on one host, while promoting interaction with another plant. At present, it is not known why bacteria are equipped with these effectors that can restrict their symbiotic interactions. One possible explanation, which would be worth investigating, is that these molecules facilitate the interactions of the given strain with legumes of the natural flora where the strain evolved, and their incompatibilities can be observed with plants with different spatial origin. The *B. elkanii* strain USDA61 is incompatible not only with *Rj4* soybeans but also with *Vigna radiata* cultivar KPS1 and different *Lotus* accessions. As with *Rj4* soybean plants, these hosts form effective symbiosis with USDA61 if its T3SS system and the above-mentioned four genes are non-functional. This similarity suggests that the proteins participate in a common mechanism contributing to nodulation restriction in these legumes. Intriguingly, the lack of another T3E Nop effector identified in the same screen as the four non-Nops, a protein of unknown function termed InnB restored the compatibility of the strain with *Vigna radiata* cultivar KPS1 but not with *Rj4* soybean, meaning that different effector proteins might be recognized by the R proteins of the two plant species. To further complicate the picture, the InnB deficient strain proved to be less efficient than the wild-type bacterium when the host was *V. mungo* (Nguyen et al., 2018). Similarly, the T3SS had a positive impact on the symbiotic efficiency of the *B. vignae* strain ORS3257 in *V. unguiculata* and *V. mungo*, but it blocked symbiosis with *V. radiata* (Songwattana et al., 2021). In line with the observations on the host-dependent effect of InnB effector, T3SS-based incompatibility of strain USDA61, with the diverse *Lotus* accessions, arrests the interaction at different developmental stages. The *L. japonicus* Gifu inhibited infection while *L. burttii* inhibited nodule maturation at the post-infection stage, whereas both in *L. burttii* and *L. japonicus* MG-20, a nodule early senescence-like response can be observed (Kusakabe et al., 2020). It was shown that NopF and NopM were the effector proteins that triggered the inhibition of infection and the nodule early senescence–like response, respectively.

In general, the T3SS of a rhizobium strain may play both positive and negative role or can be indifferent during nodulation depending on the plant partner and, thus, may determine (in)compatibility and host-range (Marie et al., 2001; Nelson and Sadowsky, 2015; Songwattana et al., 2017). For example, in the case of *S. fredii* strain NGR234 with extreme broad host range (Pueppke and Broughton, 1999), abolition of the T3SS has no effect on nodulation of *V. unguiculata*, but increased nodule number on *Pachyrhizus tuberosus* and allowed to form effective symbiosis instead of empty pseudo-nodules on *Crotalaria juncea*, while the mutant strains formed fewer nodules on *Tephrosia vogeli*, *Lablab purpureus*, and *Flemingia congesta* roots (Viprey et al., 1998; Marie et al., 2003). Similarly, it was shown that the NopC effector protein of *S. fredii* strain HH103 is beneficial for the symbiosis with *Glycine max* and *V. unguiculata* (Jiménez-Guerrero et al., 2015), but blocks nodulation with *L. japonicus* (Jiménez-Guerrero et al., 2020). The T3SS of *Mesorhizobium loti* strain MAFF303099 inhibits the interaction with *Leucaena leucocephala* (Sánchez et al., 2009) and three *Lotus* species (*L. peregrinus var. carmeli, L. subbiflorus*, and *L. halophilus*), while having positive effect on the nodulation of *L. corniculatus* subsp. *frondosus* and *L. filicaulis* (Okazaki et al., 2010). In addition, the competitiveness of the T3SS mutants against the wild-type strain also depends on the host. *L. tenuis* cultivars INTA Pampa and Esmeralda have contrasting, while *L. japonicus* MG20 has no strain preference (Sánchez et al., 2012).

#### Influence of Other Translocation Systems (T4SS and T6SS) on Symbiotic Performance

Besides T3SS, only Type IV (T4SS) and Type VI (T6SS) Secretion Systems have the ability to deliver effector proteins from bacteria into the cytosol of eukaryotic cells and, thus, to affect their interactions. Compared to T3SS, a relatively very low information is available about the potential influence of T4SS and T6SS of rhizobia on symbiotic performance and compatibility.

Most *M. loti* strains including R7A lack the genes coding for the elements and effectors of T3SS, but contain genes coding for a type 4 secretion system (T4SS) that can also deliver effectors into target cells. Interestingly, as reported on the T3SS mutants of *M. loti* strain MAFF303099, strain R7A T4SS mutants formed large and bacteroid-containing effective nodules on *Leucaena leucocephala* in contrast to the wild-type strain that could not infect the plant, were delayed in nodulation, and less competitive than the wild-type bacteria on *L. corniculatus* (Hubber et al., 2004). Based on this similarity in the phenotypes, the authors concluded that the type IV and type III system are inter-changeable and over the course of evolution, rhizobia can adopt either type. Among sinorhizobia nodulating, only *Medicago, Trigonella and Melilotus* species, the presence of T4SS is quite widespread, while T3SS have been detected in a small number of *S. meliloti* strains and no T6SS coding genes have been shown to be present in the *S. meliloti* and *S. medicae* genomes (Sugawara et al., 2013). Abolition of T4SS function in one strain of both species revealed no, as well as positive and negative, effects on the symbiosis depending on host inoculated.

The pRL1JI (pRle248a) symbiotic plasmid carries the nodulation and nitrogen fixation genes of *R. leguminosarum bv. viciae* strain 248, a symbiotic partner of *Pisum*, *Vicia*, and *Lens* species. Introduction of pRL1JI into *R. leguminosarum bv. trifolii* strain RCR5, from which its symbiotic plasmid had been cured, resulted in a strain, which could form effective symbiosis with *Vicia sativa* but failed to infect *V. hirsuta* and pea (Roest et al., 1997). A transposon insertion mutant that is able to establish nitrogen-fixing symbiosis with the two incompatible hosts led to the identification of a gene cluster that later turned out to code for the T6SS of *R. leguminosarum bv. trifolii* strain RCR5 (Bladergroen et al., 2003). In contrast to pea, the Type VI Secretion Systems of *Rhizobium etli* Mim1 (Salinero-Lanzarote et al., 2019) and *Bradyrhizobium* sp. LmicA16 (Tighilt et al., 2021) have a positive and essential role in developing effective symbiosis with *Phaseolus* and *Lupinus* species, respectively.

#### Effectors to Promote Symbiosis in the Absence of Nodulation Factors and Nodulation Factors Perception

In the majority of cases, nodule development in legume plants is initiated by the bacterial NFs that are recognized by plasma membrane receptors of the plant. It was, however, shown that certain photosynthetic bradyrhizobia, such as *Bradyrhizobium* sp. BTAi1 and ORS278, do not produce NFs because they have no *nodABC* genes, but they are able to still form nodules on the stem of certain *Aeschynomene* species, such as *A. indica, A. evenia*, and *A. sensitive*, which are exclusively nodulated by photosynthetic bradyrhizobia (Giraud et al., 2007). This surprising discovery was conformed when a *nodB* mutant of *Bradyrhizobium* sp. ORS285 could form nodules on these plants, while it could not establish symbiosis with other host species (for example, *A. afraspera*) that are stem-nodulated by both non-photosynthetic and photosynthetic strains. Searching for the genetic determinants required to induce nodule formation in the absence of NFs by transposon insertion mutagenesis has not revealed any essential genes indicating the likely redundancy of currently unknown functions involved in nodule induction (Bonaldi et al., 2010). Despite the NF independent nodule formation, nodulation of *A. evenia* requires the activity of the common symbiosis signaling pathway because if the genes coding for the Symbiosis Receptor Kinase (SYMRK), the Ca^2+^/calmodulin-dependent kinase (DMI3) or the cytokinin receptor histidine kinase (LH1/CRE1) were silenced, nodule formation was inhibited (Fabre et al., 2015).

Even NF-dependent hosts can be nodulated in the absence of NFs and NF perception. NFR1-deficient mutants of soybean varieties Enrei and Clark formed nodules when inoculated with *B. elkanii* strain USDA61 even if its NF production was abolished by a mutation in the *nodC* gene, providing the T3SS was functional (Okazaki et al., 2013). Root-hair curling and ITs were not observed in the roots and nodules formed on the NF receptor mutants, indicating that T3SS is involved in crack entry or intercellular infection. Similarly, USDA61 could form (though ineffective) nodules on NF-independent *Aeschynomene* species in a T3SS-dependent manner, however, not all the species or all ecotypes of *A. evenia* were nodulated by USDA61. These results prompted an investigation to determine whether other non-photosynthetic bradyrhizobia are able to induce nodules on these *Aeschynomene* plants, and whether such nodulation depends on the T3SS (Okazaki et al., 2016). The authors observed a whole spectrum of responses from no nodule formation through uninfected and infected ineffective nodules to nitrogen-fixing interactions. while the invasion of nodules happened through both intercellular and root hair infection.

The efficiency of the more intimate interaction between strain ORS3257 and NF-independent *Aeschynomene* accessions showed plant-determined natural variations (such as disturbed bacteroid differentiation and nitrogen fixation) and the nodule formation also depended on T3SS activity. In contrast, the genome of most photosynthetic bradyrhizobia does not contain genes coding for the T3SS and T3Es, and if it contains, as the case with strain ORS285, this translocation system is not required for NF-independent symbiosis, meaning that two mechanisms to induce nodules exists; one depends on T3SS, while the other uses a so far unknown activity. The T3SS of ORS285, however, contributes to the NF-dependent nodulation although it affects the efficiency, but not equally in all plants. The T3SS mutation had no consequences on certain NF-dependent hosts, but had positive (more nodules) or negative (less nodules) effect on others. To determine which T3Es of strains ORS3257 and USDA61 are responsible for the induction of NF-independent nodule formation on *Aeschynomene* species and the *nfr1* mutant soybean, respectively, mutants deficient in genes coding for the T3Es were inoculated on the corresponding partners. The ORS3257 mutants showed a wide variety of phenotypes (Teulet et al., 2019). One mutation increased both nodulation and nitrogen fixation, two mutations led to reduced number of nodules, some of which displayed large necrotic zones, and the nodules induced by two other mutants contained no bacteria. A strain with mutation in an effector gene termed *ernA* for “effector required for nodulation-A” and widely conserved among bradyrhizobia lost the capacity for nodule formation, not only in ORS3257 but also in strain USDA61. Introduction of the *ernA* gene into a *Bradyrhizobium* strain, which does not have an *ernA* gene and is unable to nodulate *Aeschynomene* species in an NF-independent manner, despite the presence of a functional T3SS, enabled the transconjugant to form nodules. Moreover, the nucleus targeted ErnA protein, if ectopically produced in *A. indica* roots, activated organogenesis of root- and nodule-like structures.

The Bel2-5 effector of strain USDA61 that resembles the XopD effector of the plant pathogen *Xanthomonas campestris* was shown by both loss-of-function and gain-of-function approaches to enable the *nfr1* mutant of soybean cultivar Enrei to form nodules (Ratu et al., 2021b). The same effector causes restriction of nodulation on soybeans carrying the *Rj4* allele, and it was shown by mutational analysis that most of its predicted domains were essential for both NF-independent nodulation and nodulation restriction (Ratu et al., 2021a).

#### Summary

As pathogenic bacteria, some rhizobia equipped themselves with effector molecules that can modify the functioning of plant cells and with the delivery machinery to translocate these effectors into the partners’ cells. These effector molecules sometimes facilitate more efficient interaction with certain plants and, thus, extend host-range or overcome the lack of NFs/NF recognition, but in other interactions, they trigger plant defenses, which arrest the interaction.

### Natural Variations Affecting Bacteroid Development, Persistence, and Function

#### Does Outer Membrane Composition Affect Nodule-Specific Cysteine-Rich Peptide-Induced Terminal Bacteroid Differentiation?

Legumes in the dalbergioid and inverted repeat-lacking clades of the *Papilionoideae* subfamily produce NCR and NCR-like peptides to impose terminal bacteroid differentiation on their bacterial partners (**Figure 2**). In the IRLC species, there was a positive correlation between the degree of bacteroid elongation and the number of the expressed NCRs, but the process can be affected by the inoculant. When the basal IRLC legume *Glycyrrhiza uralensis* producing seven NCR peptides is inoculated with *Mesorhizobium tianshanense* HAMBI3372, bacteroids show the signs of terminal bacteroid differentiation, such as increased cell size and decreased cell division capacity (Montiel et al., 2016), but the bacteroids of *S. fredi*i strain HH103 are not terminally differentiated in the nodules of this plant (Crespo-Rivas et al., 2016). Interestingly, the electrophoretic profile of the LPSs, especially in the O-antigen containing S(mooth)-LPS region, was markedly different from that of cultured cells when the bacteroids were isolated from *G. uralensis* nodules, whereas it was indistinguishable when bacteroids were isolated from soybean or pigeon pea. Whether this LPS modification or/and the observed decreased sensitivity toward the antimicrobial activity of cationic NCR peptides are responsible for the lack of terminal bacteroid differentiation remains to be elucidated (Crespo-Rivas et al., 2016).

#### Co-evolution of the BacA Peptide Transporters With Host Peptides

The *bacA* gene coding for a peptide transporter plays an essential role in bacteroid development and survival in those rhizobia that form nodules on NCR peptide producing IRLC legumes, such as pea and alfalfa. In contrast, the orthologous sequence is dispensable when plants such as *Lotus* not having NCRs are infected (Kereszt et al., 2011). Although the BacA proteins of different rhizobia play the same role during the terminal differentiation of bacteroids, it turned out that *bacA* genes from *S. fredii* or *R. leguminosarum bv. Viciae 3841* failed to restore the Fix^+^ phenotype of an *S. meliloti bacA* mutant on *M. sativa*, however, they allowed for further developmental progression before a loss of viability (diCenzo et al., 2017). Interestingly, the same genes could complement the mutant when *Melilotus albus* was the host and the *S. meliloti bacA* gene could complement the symbiotic defect of the *R. leguminosarum* bv. *Viciae* mutants during nodule development on pea roots. The authors showed that the *S. meliloti* BacA has rapidly diverged from the other rhizobial proteins and suggested that it most probably has evolved toward a specific interaction with *Medicago* (NCR peptides). This suggestion is in agreement with the observation that, although NCR peptides have a single origin, their evolution has followed different routes in individual IRLC legume lineages (Montiel et al., 2017).

#### Differential Role of the BclA Peptide Transporter of Bradyrhizobia

As described in a previous chapter, the Dalbergoid clade contains *Aeschynomene* species with either NF-dependent or NF-independent nodulation ability. A comparative study using the *Bradyrhizobium* strain ORS285, which can nodulate both types of plants, revealed that bacteria enter plant tissues *via* intercellular infection mechanism, and the division and development of infected founder cells give rise to the nodule tissues, where bacteria go through a terminal differentiation program (Bonaldi et al., 2011). Later it was shown that NCR-like peptides govern the terminal bacteroid differentiation in these species too, which is completed as spherical morphotype in the NF-independent species and as elongated bacteroids in NF-dependent species (Czernic et al., 2015). The differentiation of both S and E morphotype bacteroids requires the activity of the peptide transporter BclA, a homolog of *S. meliloti* BacA, both implicated in the transport of the NCR(-like) peptides, although it is required for the survival of bacteria only in the NF-independent plants (Guefrachi et al., 2015). Interestingly, *B. diazoefficiens* strain USDA110, the soybean model symbiont can nodulate the NF-dependent *Aeschynomene* species, however, the nodulation is associated with atypical bacteroid differentiation showing the signs of both the terminal and non-terminal developments and with suboptimal symbiotic efficiency (Barrière et al., 2017; Nicoud et al., 2021). The size and DNA content of the bacteroids in the *Aeschynomene* nodules and the requirement, more precisely, the indifference of the BclA protein during bacteroid differentiation were the same as in the soybean nodules, while the membrane permeability of the bacteroids is more similar to that of the ORS285 bacteroids.

#### Strain Discrimination With Nodule-Specific Cysteine-Rich Peptides

The *S. meliloti* strains A145 and Rm41 form ineffective symbiosis with *M. truncatula* cv. Jemalong (Tirichine et al., 2000; Liu et al., 2014), while their interactions with other ecotypes, such as DZA315.16 or A20, is normal. Jemalong nodules are invaded by these strains, even bacteroid differentiation and *nif* gene induction takes place at 7 days post-inoculation (dpi), but the elongated bacteroids are eliminated (14 dpi), and only saprophytic bacteria remain in the older nodules at 21 dpi (Wang et al., 2017). Surprisingly, dominant allelic variants of two genes termed *NFS1* and *NFS2* (nitrogen fixation specificity) coding for NCR peptides function as a negative regulator of symbiotic persistence (Wang et al., 2017, 2018; Yang et al., 2017). The NCRs were shown not only to be positive regulators of bacteroid development and persistence (Van de Velde et al., 2010; Horváth et al., 2015; Kim et al., 2015), but the cationic peptides also have antimicrobial activity (Van de Velde et al., 2010; Tiricz et al., 2013). Although the NCR variants from the incompatible host possess stronger antimicrobial activity than those from the compatible plants, the differences in the bactericidal activity do not correlate with and are not responsible for the *in planta* function because the responses of a compatible strain are similar. The bacterial targets of the peptides and the genetic determinants of the incompatibility in the bacteria, however, remain to be identified.

#### A Peptidase, Which Can Cleave Nodule-Specific Cysteine-Rich Peptides, Causes Incompatibility

Crook et al. (2012) described several accessory plasmids that restrict nodule development, cause impaired symbiotic nitrogen fixation, and enhance host invasion. One of these plasmids was reported to encode the HrrP metallopeptidase that is responsible for the symbiotic incompatibility with some host plants (Price et al., 2015). This enzyme was shown to cleave NCR peptides *in vitro*, although with different efficiency, suggesting some level of NCR peptide substrate selectivity. Bacteroids in the nodules—that are much smaller than normal, most probably because of the loss of meristem persistence—on the incompatible host plant develop normally and express the *nif* genes but in older cells, they appear to fragment and to degenerate. In addition, the saprophytic rhizobial population in both compatible and incompatible nodules is significantly larger. One possible explanation for these phenotypes is that the peptidase cleaves NCR peptides/peptide variants, both those that are required for bacteroid persistence and those that have antibacterial activity. This cleavage activity might be more efficient on the peptides of the incompatible ecotypes leading to bacteroid senescence. On the other hand, cleaving the cationic peptides that would restrict the cheaters might increase the saprophytic population.

#### Host-Specific Regulation of Nitrogen Fixation Genes in Clovers

Strains of *R. leguminosarum* bv. *trifolii* (such as ICC105), able to form effective nodules on *Trifolium ambiguum* (Caucasian clover), form bacteroid containing but ineffective nodules on *T. repens* (white clover), whereas strains (for example, NZP514) that form effective nodules on white clover usually do not nodulate Caucasian clover. It was shown (Miller et al., 2007) that the *nifHDKEN*, *fixABCX*, and, probably, the *NifBfdxNfixU* operons of strain ICC105 are not induced in the white clover nodules that were not caused by the lack of *nifA* expression or NifA function. Rather, it is supposed that the intergenic region between the *nifH* and *fixA* genes might be bound not only by the canonical transcription factors NifA and RpoN, but also by additional host-specific regulatory elements. This proposed protein might bind upstream of the NifA binding sites either in response to or in the absence of a host-specific signal and interacts with NifA to prevent it from activating the expression of the *nif/fix* operons.

#### Summary

Certain host plants impose terminal bacteroid differentiation on their rhizobial partners with the help of NCR(-like) peptides produced in the infected nodule cells. The NCRs are required not only for bacteroid differentiation, persistence, and functioning, but are also used for strain discrimination and elimination. Bacteria might counteract the effects of NCRs by producing a modified outer membrane or a peptidase cleaving the peptides. Some of these peptides possess antibacterial activity, which is attenuated by the BacA/BclA peptide transporters to a different extent in different hosts that show only limited interchangeability between bacteria infecting legumes with diverse peptide profiles. It is not known whether NCRs or other factors are responsible for the host-specific activation of the nitrogen-fixation genes in nodules of clovers.

## Partner-Dependent Manifestation of Mutations

The majority of the genes required for the development and function of nitrogen-fixing nodules were identified by forward genetics studies when the failure of the mutants in establishing effective symbiosis indicated their essentiality in the interaction. Intriguingly, there are cases, apart from bacterial T3SS effectors and ETI elements, when a mutation manifested in the arrest of the interaction at a certain developmental stage with one partner, whereas in no disturbance or halt in another stage with another partner(s).

### Host Plants Put Different Levels of Stress on Their Partners

The production of NFs and exopolysaccharides are controlled by complex regulatory networks including the stringent response, which induces a physiological change in response to adverse growth conditions/stress and can also control bacterial development, virulence using guanosine tetra- and penta-phosphate (ppGpp) as effector molecules (Wells and Long, 2002). The *relA*-deletion mutant of *S. meliloti* incapable of ppGpp synthesis fails to nodulate *M. sativa* because of the early arrest in IT development and meristem formation, but successfully infects *M. truncatula* although bacteroids have disorganized positioning within nodule cells and reduced nitrogen-fixation capacity in this host (Wippel and Long, 2019). The mutant seems to produce NFs and EPS in a higher amount than the wild-type cells and these elevated levels might be inhibitory for alfalfa and neutral or even stimulant in barrel medic. Transcriptomic changes in the mutant upon luteolin induction also pointed to other stress regulatory processes, such as osmoregulation, that might also affect the infection process. In the nodules of *M. truncatula*, the number of differentially expressed bacterial genes are rather low, however, the upregulation of numerous transcripts related to metabolism and transcriptional regulation depends on RelA, indicating that full metabolic adaptation to the nodule environment and, as a direct or indirect consequence, the normal bacteroid organization and nitrogen fixation might require RelA function.

Mutation in another stress-related gene of *S. meliloti*, *typA*, coding for a ribosome-binding GTPase acting at the level of protein synthesis, was shown to be involved not only in stress adaptation, but to be required for the establishment of nitrogen-fixing symbiosis on certain *Medicago* hosts (Kiss et al., 2004). The mutant formed efficient nodules on different *M. sativa* cultivars, with only ∼20% decrease in shoot dry weight of some lines and with *M. truncatula* ecotype DZA315.16. In contrast, it formed Fix^–^ nodules on ecotypes Jemalong and F83005, however, the nodules on the former were small and devoid of bacteroids, while no obvious differences in nodule occupancy between the wild-type and mutant bacteria-induced F83005 nodules could be observed. The TypA and related proteins in other bacteria are required for the adaptation to several stresses under different conditions. Rhizobia must adapt to ever-changing physiological conditions such as pH, ion composition, osmotic concentration, oxidative stress, or plant defense reactions during nodule invasion and bacteroid differentiation. These conditions, which are most probably different in each host plant, may explain why TypA is not required equally on the investigated hosts.

### The Severity of a Carbon Metabolism Defect Depends on the Host Plant

Energy and carbon sources for bacteria in the nodule cells are provided in the forms of the TCA-cycle intermediary C4-dicarboxylates, succinate, fumarate, and malate that necessitate the activity of gluconeogenesis to generate intermediates for growth and metabolism. The first step of gluconeogenesis, the decarboxylation and phosphorylation of the TCA-cycle intermediate oxaloacetate is catalyzed by the phosphoenolpyruvate carboxykinase (PEPCK) enzyme. The effect of a mutation in the *pck* gene coding for PEPCK in *S. fredii* strain NGR234 on nodule development and nitrogen fixation was shown to depend on the host inoculated with the mutant (Osteras et al., 1991). The determinate nodule-forming plants, *Macroptilium atropurpureum* and *V. unguiculata* formed fewer nodules when inoculated with the mutant as compared to the wild-type that provided very low or no gain in plant biomass attributable to the symbiosis. On *Leucaena leucocephala* forming indeterminate nodules, the mutant induced an excess number of nodules that had 60% of wild-type efficiency of symbiotic nitrogen fixation. In all nodules harboring the mutant, the number of infected cells was lower compared to that observed in NGR234 induced nodules. One possible explanation for the variability of symbiotic efficiency is that different carbon source availability might be provided by a given plant during the invasion, bacteroid development, and nitrogen-fixation that would determine the relative importance of the bacterial gluconeogenesis pathway.

### Plant Mutations Cause Bacteroid Senescence Only for Certain Strains

The forward genetic screen of *M. truncatula* Tnt1 insertion mutants using *S. meliloti* strain 1021, as inoculant identified the *npd1-1* mutant (line NF4608), which formed Fix^–^ nodules, arrested at an early developmental phase, just after bacteria are released from the ITs (Pislariu et al., 2019). The released rhizobia start to elongate but fail to differentiate into functional, nitrogen-fixing bacteroids, rather, they go through a degradative senescence process most probably caused by the activation of plant defense responses. Interestingly, strain Rm41 induces functional, nitrogen-fixing nodules that can support plant growth as in wild-type plants. The mutation abolishes the function of a symbiotically expressed gene termed *NPD1* (Nodule-specific PLAT Domain) coding for a small protein localized in the endoplasmatic reticulum (ER) and symbiosomes, but not co-localized with Golgi. It is supposed that NPD1 with other ER-localized proteins suppresses rhizobium induced plant defense reactions. How and why the hosts discriminate against bacterial strains remains to be elucidated.

The *L. japonicus apn1* (*sym104*) mutants, when inoculated with *Mesorhiozobium loti* strain TONO, display severe nitrogen deficiency symptoms and form small white nodules, in which the early senescence of nodule cells and the bacteroids therein can be observed (Yamaya-Ito et al., 2018). This senescence was manifested in a decrease in bacteroid density, abnormal enlargement, and irregular shapes of symbiosomes, the presence of lytic vacuoles, and disintegration of infected cells. Interestingly, not all the *M. loti* strains induce Fix^–^ nodules on the mutant plants; out of the nine strains tested five bacteria, including strain MAFF303099, formed effective pink nodules. The mutations in *Lotus* disrupt a symbiosis-specific late nodulin gene coding for a secreted aspartic peptidase enzyme termed ASPARTIC PEPTIDASE NODULE-INDUCED 1 (APN1). Random mutagenesis of strain TONO identified the causal gene termed *DCA1* (Determinant of nitrogen fixation Compatibility of APN1) causing the incompatibility that encodes an active autotransporter, one of the proteins also known as the Type V protein secretion system found in Gram-negative bacteria (Shimoda et al., 2020). The protein contains an N-terminal secretion signal peptide, a C-terminal autotransporter β-domain, and 43 divergent glycine-rich repeats of 44–53 amino acids. Autotransporters cleave a part of their protein known as the passenger domain and transport it through the outer membrane to the outside of the cell and the whole length of this passenger domain with all glycine-rich repeats is required for its activity. It was shown that the APN1 protein can degrade the DCA1 protein under the acidic conditions required for the activity of aspartic peptidases. The phenotypic dissimilarity caused by the different strains producing orthologous, basically identical autotransporter is determined by the expression differences of the gene in the bacteria, i.e., *DCA1* promoter-GUS activity in wild-type and *apn1* mutant nodules was negligible in strain MAFF303099 and high in strain TONO. It is proposed that strain TONO produces a higher amount of the effector protein than MAFF303099 that is degraded by the APN1 aspartic peptidase in the wild-type plant. In the mutant plants, however, the effector is not degraded and is able to induce the expression of cysteine protease genes leading to suppression of nodule maturation and initiation of senescence, while the amount of DCA1 from MAFF303099 is not enough for the induction.

#### Summary

Bacteria face diverse levels of stresses and defense responses, as well as metabolite availability and composition, in different wild-type and mutant plants. The ability of wild-type and mutant bacteria to respond and adapt to those conditions is not the same in different strains, and that is manifested in phenotypic variations. These phenotypic variations will allow us to reveal what causes the differences.

## Conclusion and Perspectives

Exploring natural variations has already facilitated the recognition of important determinants of symbiotic nodule initiation, development, and function, such as the identification of the LysM2 domain of NFR5 as a potential binding site for the bacterial NFs or recognition of the role of T3SS, Type3 effectors, and ETI in partner selection or revealing that NCR peptides, not only direct the terminal differentiation and persistence of bacteroids, but also participate in partner discrimination and selection. These examples as well as the results with mutants showing multiple phenotypes depending on the hosts suggest that it is worth to reinvestigate the already existing mutants with other symbiotic partners and identify the genetic determinants behind the phenotypic differences in both wild-type and mutant interactions. The agricultural productivity of legume crops depends on their interaction, i.e., (in)compatibility with the rhizobia resident or introduced as inoculum in the soils that is also affected by the competition of the different strains. Similarly, engineering nitrogen-fixing non-legume crops in the future, as well as their cultivation on the field, requires the knowledge of the details and fine-regulation of the symbiotic process. The results of studies on natural variations will identify the determinants of symbiotic (in)compatibility and reveal the fine details that exist in the regulation of the interaction, thus, may contribute to the implementation and realization of the plans aiming at the development of novel crops for sustainable agriculture.

## Author Contributions

TW, BB, and SK collected, collated, organized the reviewed publications, and participated in the writing of the manuscript. AK conceived the topic of the review, supervised the co-authors’ work, and wrote the manuscript. All authors contributed to the article and approved the submitted version.

## Conflict of Interest

The authors declare that the research was conducted in the absence of any commercial or financial relationships that could be construed as a potential conflict of interest.

## Publisher’s Note

All claims expressed in this article are solely those of the authors and do not necessarily represent those of their affiliated organizations, or those of the publisher, the editors and the reviewers. Any product that may be evaluated in this article, or claim that may be made by its manufacturer, is not guaranteed or endorsed by the publisher.

## Footnotes

1 Luu, T., Ourt, A., Pouzet, C., Pauly, N., and Cullimore, J. (2022). *A Newly-Evolved Chimeric Lysin Motif Receptor-Like Kinase in Medicago truncatula spp. Tricycla R108 Extends Its Rhizobia Symbiotic Partnership*.
